# Where you live shapes who you are: morphological changes in urban *Triatoma infestans*

**DOI:** 10.3389/finsc.2025.1593921

**Published:** 2025-06-02

**Authors:** Romina V. Piccinali, Julieta Nattero, Florencia Cano, Paz Sánchez-Casaccia, Ana L. Carbajal-de-la-Fuente

**Affiliations:** ^1^ Laboratorio de Eco-Epidemiología, Departamento de Ecología Genética y Evolución, Facultad de Ciencias Exactas y Naturales, Universidad de Buenos Aires, Ciudad Autónoma de Buenos Aires, Argentina; ^2^ Instituto de Ecología, Genética y Evolución (IEGEBA), CONICET-Universidad de Buenos Aires, Ciudad Autónoma de Buenos Aires, Argentina; ^3^ Programa de Control de Enfermedades de Transmisión Vectorial, Ministerio de Salud, San Juan, Argentina; ^4^ Centro Nacional de Diagnóstico e Investigación en Endemo-Epidemias (CeNDIE), Administración Nacional de Laboratorios e Institutos de Salud “Dr. Carlos Malbrán” (ANLIS), Ciudad Autónoma de Buenos Aires, Argentina; ^5^ Consejo Nacional de Investigaciones Científicas y Técnicas (CONICET), Ciudad Autónoma de Buenos Aires, Argentina

**Keywords:** triatomines, urbanization, Chagas, geometric morphometrics, linear morphometrics, size, shape, Argentina

## Abstract

**Introduction:**

Urbanization has transformed landscapes, driving ecological and morphological changes in insects. Chagas, traditionally a multidimensional rural problem, is increasingly reported in urban areas. *Triatoma infestans*, the primary vector of Chagas disease in the Southern Cone, has been reported in urban centers of San Juan, Argentina, for decades. Using morphometric and colorimetric analyses, we assess how urbanization influences the morphology and coloration of *T. infestans*.

**Materials and Methods:**

A total of 105 adults from five urban and one rural population of San Juan were analyzed.Wings, pronota, heads, and legs were measured and compared between populations and sexes. Principal Component and Canonical Variate Analyses were performed to assess shape variations. Kruskal-Wallis and Wilcoxon tests, and linear models examined size differences. Colorimetric analyses searched for wing and connexivum color differences between individuals.

**Results:**

Multivariate analyses revealed significant morphological differentiation of wing, pronotum, and head shapes, primarily distinguishing the rural Valle Fértil from urban populations. Centroid size analyses indicated that rural individuals exhibited larger body structures, a pattern generally consistent across sexes. Furthermore, leg morphology also varied, with Valle Fértil insects possessing greater femur length and width compared to their urban counterparts. No significant color differences were found across populations or sexes.

**Discussion:**

Urban *T. infestans* exhibit size reductions, aligning with Schofield’s simplification hypothesis and possibly influenced by the Urban Heat Island effect. Shape changes, more pronounced in wings and pronota, suggest other influences beyond the rural-urban gradient, potentially including developmental plasticity, flight demands, and genetic drift. These findings underscore the need for urban-specific Chagas disease control strategies and further research on the evolutionary dynamics of *T. infestans* in urban environments.

## Introduction

Urbanization is a process where dense settlements of buildings, roads, and supporting infrastructure are created by humans (1). Eighty-one percent of the population of Latin America and the Caribbean lives in cities (2), with urban built-up densities higher than in North America or Europe (3). In addition, Latin America is the region with the greatest proportion of population concentrated in megacities (cities with 10 million inhabitants or more): 14.2% of the population lived in six cities by 2018 (2).

Urban areas have shaped the land surface and created new environmental conditions, provoking effects on the ecology of the organisms that live in cities and their associated morphological traits (4). Insects are not an exception to this observation and have changed to live in urban settings by using phenotypic plasticity strategies (5, 6) or evolving new traits (7). For instance, the London Underground mosquito (*Culex pipiens molestus*) has genetically diverged from its surface counterpart to adapt to subterranean conditions, including altered feeding and mating behaviors, to live in human created environments like cellars, basements and subways all over the world (8, 9). Using long-term observational data, Merckx et al. (10) found that Nordic populations of the butterfly *Pieris napi* and the moth *Chiasmia clathrata* have longer flight seasons and end later in most cities, suggesting a difference in the timing of diapause induction in urban areas compared with neighboring rural populations. Additionally, invasive Argentine ants (*Linepithema humile*) have formed supercolonies in urban areas, exhibiting social flexibility and enhancing their survival in fragmented habitats (11). Color changes have also been associated with urbanization in insects, with an increase in melanic morphs detected in urban or industrialized areas (12). These examples highlight how urban environments could act as strong selective forces, driving rapid evolutionary changes in insects.

Chagas is one of the most important concerns of public health in Latin America, being one of the most expanded endemics in the continent. It is a complex health problem with multiple dimensions including biomedical, cultural, educational, polítical, económical, environmental and social aspects (13). For many years, Chagas disease has been considered an exclusively rural issue, primarily affecting people in impoverished areas with limited access to sturdy housing, utilities, and healthcare services. In the absence of vaccines against *Trypanosoma cruzi*—the parasite responsible for Chagas disease—control efforts for vector-borne transmission have focused on spraying pyrethroid insecticides to target rural populations of insect vectors, which include several species from the Triatominae family (14). However, global trends in human population growth and urbanization have led to a rising number of incidents involving emerging infectious diseases. In urban settings, the coexistence of humans with animals and insect vectors creates favorable conditions for the spread and transmission of zoonotic diseases (15). A recent review identified 18 species of triatomines from the genera *Panstrongylus*, *Rhodnius*, and *Triatoma* as being associated with urban infestations across the American continent, spanning from Argentina to the southern United States (16). Moreover, urban infestations have been on the rise since the 1990s, becoming increasingly widespread and more frequently reported over the past three decades. *Triatoma infestans* remains the principal vector of Chagas disease in the Southern Cone of South America. Despite a significant reduction in its historical geographic distribution due to control efforts under the Southern Cone Initiative, the species continues to be present in certain regions of Argentina, Bolivia, and Paraguay, where it poses a persistent socio-environmental health challenge (17). In Argentina, although some provinces are still at risk of vectorial transmission, available epidemiological data are outdated and mostly derived from rural entomological surveys. Recent analyses that incorporate diverse data sources—beyond official surveillance—indicate that *T. infestans* has been detected in 92% of the national jurisdictions (22/24) since 2000, with most records concentrated in the Dry Chaco and Monte eco-regions, where the species has been historically registered (18, 19). Health authorities in the Argentine province of San Juan have reported the presence of this species in urban environments for several decades, including the capital city. An entomological survey conducted in 2016 across 2,427 houses in San Juan city and neighboring urban areas (Gran San Juan) revealed an infestation rate ranging from 2% to 20% (20). In 2017, hundreds of reports of *T. infestans* being present in homes were made by residents, and provincial health authorities confirmed cases of acute vector-borne transmission of *Trypanosoma cruzi* between 2016 and 2020 (21). A study conducted in the Rawson Department, over 75 blocks and 1,784 houses found a total of 958 *T. infestans* adults and nymphs 7.6% of them infected with *T. cruzi* in 2017 (20).

Morphometric techniques provide objective, quantifiable data on morphology, enabling researchers to address diverse questions about triatomine species. For example, differentiation among closely related species has been evaluated within the *T. sordida* subcomplex (22) and the *T. rubrovaria* subcomplex (23), as well as between *Rhodnius neglectus* and *R. prolixus* (24), *Mepraia* sp*inolai* and *M. gajardoi* (25), and between *T. arthurneivai* and *T. wygodzinskyi* (26). Other studies have analyzed population differentiation within *T. dimidiata* (27), *T. brasiliensis* (28), and *T. infestans* (29, 30). Finally, recent research has explored morphological differences as potential adaptations to different environments in species such as *T. dimidiata* (31, 32), *R. ecuadoriensis* (33) and *T. guasayana* (34).

The present study is part of a broader research project developed through an inter-institutional collaboration involving municipal, provincial, and national health authorities, academic researchers, university students, and local communities. This project seeks to promote an integrated approach to understanding and addressing urban Chagas disease and the main aspects we aim to explore in the present study are: Does urbanization affect the adult morphology of *T. infestans*? If so, how does it influence traits such as wing, pronotum, head, and leg size and shape? Are there sex differences in phenotypic plasticity? Are color differences associated with urbanization? To answer these questions, we analyzed insects collected in San Juan Province from areas with different degrees of urbanization, using linear and geometric morphometrics as well as colorimetric analyses.

## Materials and methods

### Study areas

This study was carried out with insects collected in two areas of the Argentine Province of San Juan. One is the urban agglomerate known as Gran San Juan (31°32′ S, 68°32′ W), which includes the Departments of Capital, Rivadavia, Rawson, Chimbas, Pocito, and Santa Lucía. It is the main urban center of the San Juan Province and accounts for 73.8% of the province’s total population (35). It is located within the Tulum-Ullum-Zonda valley, an oasis under irrigation as a result of the systematization of surface river courses and subsoil extraction with significant human occupation and development of intensive agriculture. Although the main economic activity of the province is currently metal mining, its wine, olive, horticultural and tourism production also stands out. The climate is desertic with summer concentrated precipitation (less than 250 mm, dropping to 30 mm in some valleys) and a mean annual temperature of 17.9°C (BWk in the Köeppen-Giger classification scale) with an annual temperature range from 3.3°C to 32°C due to continentality and aridity (36, 37). The wild environment surrounding the metropolitan area belongs to the Monte ecoregion, a tall steppe (1–3 m) dominated by *Larrea* sp., *Bulnesia retamo*, and other species, typical of the *jarillal* community. It grows on well-drained soil in valleys, while on mountain slopes, shorter shrubs and columnar cacti prevail. In cities located in arid areas such as San Juan, where the richness of native tree flora is very low—between three to five species, with small leaves and limited canopy development—their presence in the urban forest is greatly reduced. However, there are more than 90 tree species in total (38), with two exotic species, *Morus nigra* and *Platanus acerifolia*, dominating in terms of presence and coverage (39).

The other area comprises the village called Villa San Agustín (30°40′ S, 67°26′ W) in the Department of Valle Fértil, which lies on the eastern slope of the Sierras Pampeanas; and along a valley covered by abundant vegetation. Its dynamics as a population center are in the process of urban consolidation, while the system as a whole maintains a mainly rural character. The climate is semiarid steppe with higher precipitation (more than 250 mm) distributed along the summer months (BSk in the Köeppen-Giger classification scale (36)). The mean annual temperature is 16.9°C, with an annual temperature range from 4.4°C to 29.1°C (40). It belongs to the Dry Chaco ecoregion, whose vegetation is xerophytic forests, comprising an upper tree stratum of moderate height; a middle stratum of shrubs, often forming a dense thicket; and a lower stratum of herbaceous plants, mainly annuals and cacti, which cover the ground in a discontinuous manner.

We categorized the insects collected in Gran San Juan as “urban” and those collected in Valle Fértil as “rural”, being aware that the urban-rural dichotomy is not as straightforward as it seems, as there is a continuum between both extremes (41).

### Insect collection

**Figure 1:**
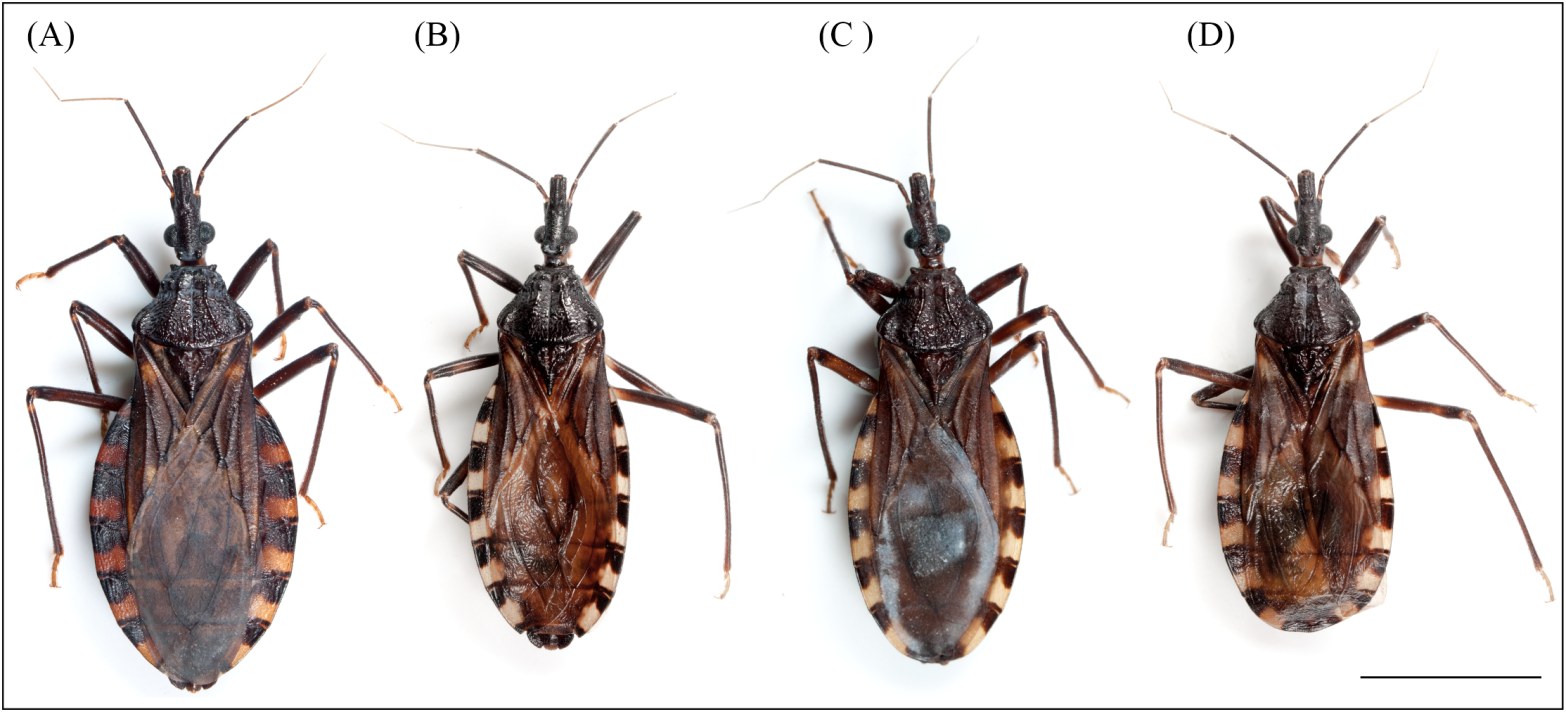
Rural female **(A)**, urban female **(B)**, rural male **(C)** and urban male **(D)** of the collected *T. infestans* from San Juan, Argentina. Scale bar: 21.8 mm.

We analyzed a total of 105 adults of *T. infestans*, 52 females and 53 males collected in 2018 and 2020 (**Figure 1**, **Supplementary Material 1**). Urban *T. infestans* (N = 87) were spontaneously collected by local residents at their homes and taken to the San Juan Program of Control of Vector-Borne Diseases notification center, due to the complexity of implementing the traditional vector control program’s entomological searches in urban areas (42). They were sorted by the provincial department where they were found and preserved in 70% ethanol. Rural *T. infestans* adults (N = 18) were collected by trained personnel of the San Juan Program of Control of Vector-Borne Diseases following the recommendations of the “Guide for vector control of Chagas disease” of the National Health Ministry (43). All the insects were identified following Lent and Wygodzinsky (44) and the Catalog of Argentine Triatomines (19) and kept in jars with 70% ethanol until their processing.

### Photographs

Heads and pronota were mounted in a petri dish, using glass beads as support. Right forewings or hemelytra (from here on “wings”), along with the first and second right legs, were carefully removed with entomological forceps and placed in petri dishes containing 70% ethanol. The four structures of each specimen were individually photographed under a Leica M205A microscope equipped with a Leica DF295 camera at the *Museo Argentino de Ciencias Naturales “Bernardino Rivadavia”*. All images included a reference scale.

### Geometric morphometrics

We recorded ten landmarks for wing venation origins and intersections, six landmarks for the pronotum, and six landmarks for the head (**Supplementary Material 2**) of each individual using the software TPSdig2 ver. 2.31 (45). Morphological analyses were performed separately for females and males due to the sexual dimorphism present in *T. infestans* (29). Wing, pronotum, and head shape variables and centroid sizes (CS) for each triatomine were obtained using the generalized Procrustes analysis superimposition algorithm, implemented in the R package Morpho 2.12 (46). The multivariate regression of Procrustes coordinates on CS was significant for both sexes for the wing (females: R² = 0.056, p = 0.006; males: R² = 0.051, p = 0.016), for the males’ pronotum (females: R² = 0.039, p = 0.084; males: R² = 0.070, p = 0.005), and for the females’ head (females: R² = 0.107, p = 0.001; males: R² = 0.02, p = 0.408). However, because the allometric effects were small, analyses were performed using the original Procrustes coordinate values. The presence of outliers was checked with geomorph 4.0.8 (47). One male from Rawson and another from Chimbas were considered outliers and were excluded from all the wing analyses. In addition, one female from Rawson was excluded from head analyses.

Principal Component Analyses (PCA) and Canonical Variate Analyses (CVA) were performed on the covariance matrix derived from the aligned shape data to reduce dimensionality and summarize the main patterns of variation in wing, pronotum and head shapes. Mahalanobis distances were also estimated to assess pairwise differences in shape among populations. All the analyses were made with Morpho 2.12. We applied Kruskal-Wallis tests and *post hoc* pairwise Wilcoxon rank sum tests to search for differences in mean CS among populations for each body structure. P-values were adjusted using the Holm’s method (48).

### Linear morphometrics

The length and width of the first (LLI and WLI) and second femur of the right legs (LLII and WLII) were measured using TPSdig2 ver. 2.31 (**Supplementary Material 2**). Linear models were applied to each leg measurement, with sex and population as fixed factors, using R software (49). Assumptions of normality and homoscedasticity were analyzed with the diagnostics from the R package DHARMa (50).

### Colorimetric analyses

The best photographs of three males and three females from each population were chosen to analyze differences in wing and connexivum color. The complete wings were cropped to remove the background, while a rectangle of 1.5 × 18 mm from the right side of the connexivum was obtained, as in Nattero et al. (51). The colorimetric analyses were performed separately for each structure using the web software Image Color Summarizer (52). Pixels were clustered into two groups based on luminance, chroma, and hue. We obtained the average value of each red, green, and blue (RGB) component for each cluster per individual (six variables in total). K-means clustering analyses were used to define the number of colorimetric groups present in the six sampled *T. infestans* populations for wings and connexiva. The optimal number of clusters for each dataset was determined using the multiple indexes implemented in the R package NbClust (53).

## Results

### Shape

**Figure 2:**
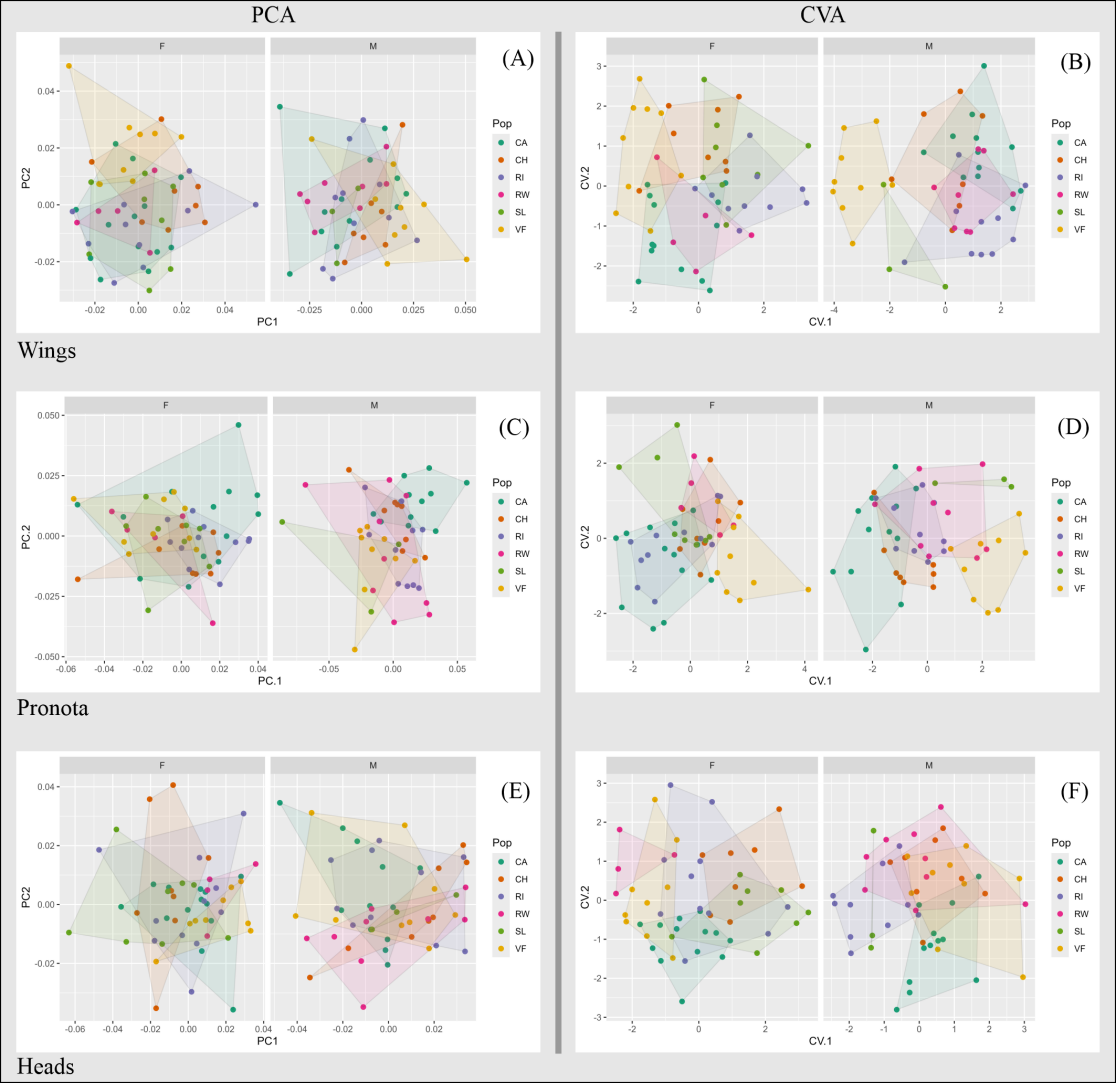
Scatterplots of the first two PC scores (left) and CV scores (right) for wing **(A, B)**, pronotum **(C, D)** and head **(E, F)** shapes of urban and rural *T. infestans* individuals from San Juan. F, females; M, males; CA, Capital; CH, Chimbas; RI, Rivadavia; RW, Rawson; SL, Santa Lucía; VF, Valle Fértil.

The first two axes of the PCA explained 47.49% of wing shape variation in females and 49.23% in males. Along PC1, variation in female wings was primarily associated with the positions of landmarks 3, 4, and 5, while variation along PC2 was most pronounced at landmarks 6, 7, and 8 (**Figure 2A**, **Supplementary Material 3**). Interestingly, rural wings from Valle Fértil tended to exhibit wider shapes. In contrast, male wings showed less shape variation than female wings. Along PC1, male wings exhibited broad variation at landmarks 7 and 8, and along PC2 at landmarks 8, 9, 10, and 3 (**Figure 2A**, **Supplementary Material 3**). Individuals from Valle Fértil tended to appear on the right side of the graph, representing less elongated shapes (**Figure 2A**).

The first two axes of the CVA explained 62.62% and 80.06% of female and male wing variation, respectively (**Figure 2B**, **Supplementary Material 4**). Cross-validation reclassification values were low for the overall analysis (females: 28.30%, males: 18.37%), while the populations with higher reclassification values were Rivadavia for females (40.00%) and Valle Fértil for males (77.77%). Mahalanobis distances were significant primarily in comparisons between rural and urban populations for both sexes; females from Capital also differed significantly from females in other urban populations such as Chimbas, Rivadavia, and Santa Lucía (**Table 1**). Additionally, females from Chimbas differed significantly from those in Rivadavia.

**Table 1:** Mahalanobis distances between rural and urban populations of *T. infestans* for each body structure.

POPS	FEMALES			MALES		
	WING	PRON	HEAD	WING	PRON	HEAD
VF-CA	**2.72**	**2.70**	2.02	**4.65**	**4.08**	1.87
VF-CH	**2.92**	1.79	3.10	**3.65**	**3.17**	1.31
VF-RI	**3.56**	**2.52**	2.07	**4.69**	**2.89**	**2.54**
VF-RW	2.98	2.20	2.20	**4.42**	2.25	1.93
VF-SL	**3.23**	**2.65**	**3.38**	3.43	2.35	2.81
CA-CH	**2.83**	1.95	**2.52**	1.96	1.78	1.98
CA-RI	**2.81**	1.05	1.70	2.16	1.78	2.22
CA-RW	2.03	2.26	2.87	1.91	**2.52**	2.21
CA-SL	**2.95**	1.62	**2.57**	3.91	**4.16**	2.54
CH-RI	**2.83**	1.33	1.67	2.34	1.33	2.02
CH-RW	3.01	1.55	**3.75**	2.00	1.84	1.31
CH-SL	2.86	1.45	2.00	3.51	**3.63**	2.50
RI-RW	2.59	1.92	2.67	2.00	1.05	2.08
RI-SL	2.48	1.52	2.11	3.73	2.98	1.76
RW-SL	2.88	1.55	**4.10**	3.44	2.08	2.33

Shaded cells: urban-rural comparisons. POPS, populations.

PRON, pronotum; CA, Capital; CH, Chimbas; RI, Rivadavia; RW, Rawson; SL, Santa Lucía; VF, Valle Fértil.

Values in bold are significant after Holmes’ correction.

The PCA explained 63.03% of female and 65.47% of male pronotum variation (**Figure 2C**). The first component primarily reflected changes at landmarks 2, 3, 5 and 6 in both sexes (**Supplementary Material 3**). The second component was associated with changes at landmarks 6 and 2 in females, and at landmarks 2 and 3 in males (**Supplementary Material 3**). The first two components of the CVA explained 81.07% of female pronotum variation and 88.23% of male shape variation. Most rural individuals from Valle Fértil grouped separately from the rest, as did Capital males and Santa Lucía females (**Figure 2D**). Global reclassification values were similar to those for wing shape in females (20.37%) but were higher in males (31.37%). The highest reclassification values were obtained for Valle Fértil females (55.56%) and for males in Valle Fértil (55.55%) and Chimbas (62.50%). Mahalanobis distances were significant in 6 out of 10 rural-urban comparisons, and for males, significant differences were observed between Capital and Rawson, Capital and Santa Lucía, and Santa Lucía and Chimbas (**Table 1**).

The first two components of the PCA for head shape captured 51.51% of female variation and 47.60% of male variation (**Figure 2E**). Along the first axis, landmarks 4 and 5 (between the anteclypeus and postclypeus) and landmarks 1 and 8 (postocular region), defining more or less elongated head shapes, were the most variable for both sexes (**Figure 2E**, **Supplementary Material 3**). Landmarks 1 and 8 were also the most modified for females along the second PCA axis, resulting in a more or less wide postorbital region. For males, changes along this axis affected landmarks 3, 4, and 6, altering the shape of the antennal tubercle and the ante-postclypeus regions (**Supplementary Material 3**).

The first two axes of the CVA explained 75.74% of female head variation. Groups overlapped on the plot (**Figure 2F**), and the overall reclassification rate, as with previous morphological structures, was low (20.75%). The best-reclassified population was Chimbas (37.50%). Variation among males along the first two axes comprised 74.27% of the total variation. The overall reclassification rate was 29.41%, with Capital as the best-differentiated population (58.00%). Mahalanobis distances for females were significant for four urban-urban and two urban-rural comparisons (**Table 1**). Capital was different from Chimbas and Santa Lucía, and these two last populations differed from Rawson and Valle Fértil. For males, the only significant comparison was between Valle Fértil and Rivadavia.

### Centroid size

We found significant differences in CS among populations for female wings (Chi-squared: 22.693, df = 5, P-value = 0.00039), pronotum (Chi-squared: 27.053, df = 5, P-value = 0.0000557), and head (Chi-squared: 23.897, df = 5, P-value = 0.0002273). In males, wing CS (Chi-squared: 14.955, df = 5, P-value = 0.01056) and head CS (Chi-squared: 15.306, df = 5, P-value = 0.009131) varied significantly among populations.

**Figure 3:**
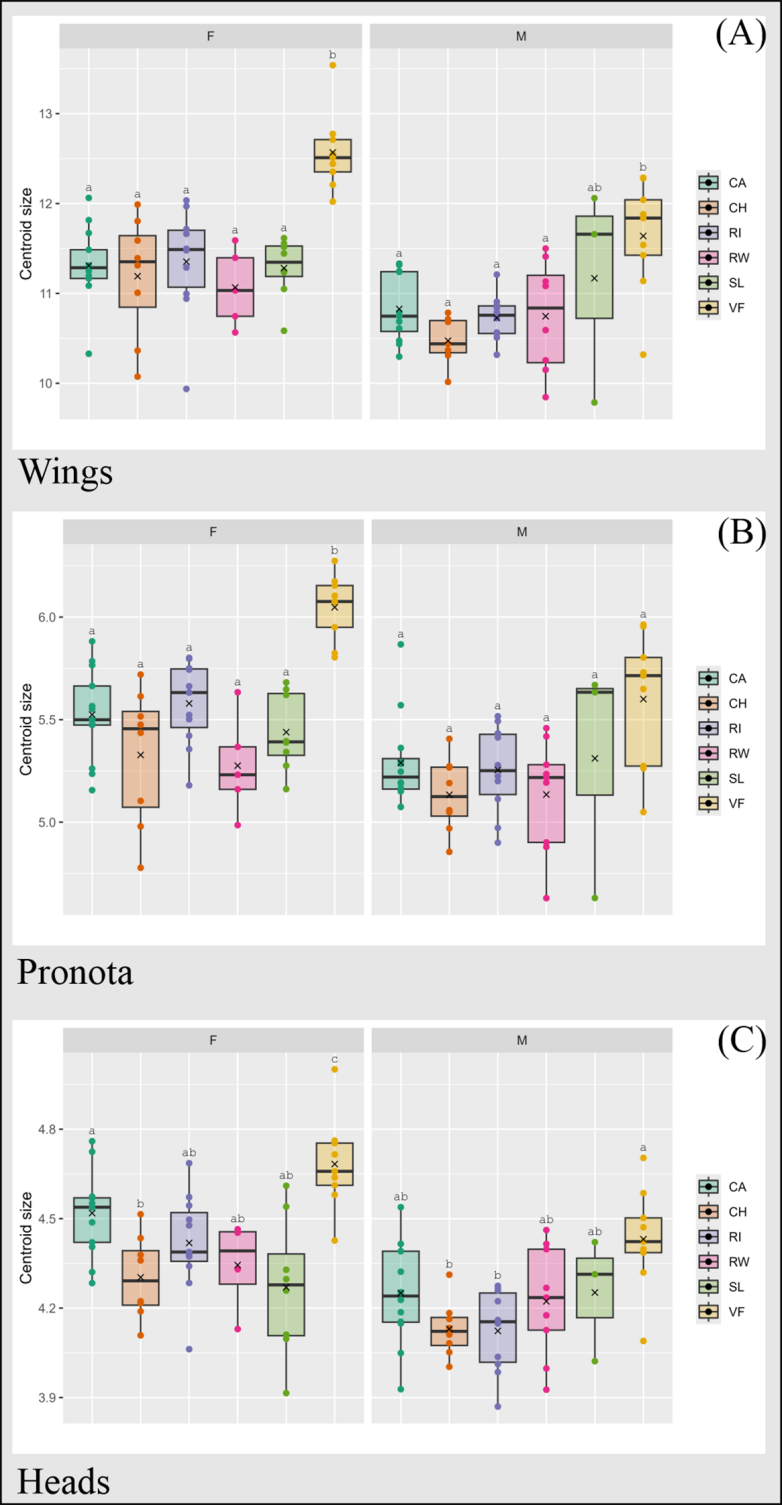
Centroid size (CS) boxplots for wings **(A)**, pronota **(B)** and heads **(C)** of urban and rural populations of females (F) and males (M) of *T. infestans* from San Juan. CA, Capital; CH, Chimbas; RI, Rivadavia; RW, Rawson; SL, Santa Lucía. VF, Valle Fértil. Different letters (a–c) indicate different mean values. Thick line: median value. X: mean value.

Mean female wing, pronotum, and head CS from the rural Valle Fértil population were higher than those from all urban populations (**Figure 3**). No differences were found among urban populations, except for head CS, which was higher in Capital than in Chimbas (**Figure 3**).

Male wing CS followed the same pattern as mean female wing CS, except for the comparison between Valle Fértil and Santa Lucía. Pairwise comparisons of head CS showed two significant differences, with higher CS in Valle Fértil than in Rivadavia and Chimbas.

### Linear measurements

The linear models evaluating the different legs´ measurements as a function of population and sex revealed significant effects for both factors and their interaction (**Table 2**). The R² values indicated that the models explained between 30.2% and 42.1% of the variance in legs´ measurements, with adjusted R² values between 21.7% and 35.2%. For the population of Valle Fértil, the effects were significant and positive compared to Capital for both LLI (Estimate = 0.37, p < 0.001) and WLI (Estimate = 0.07, p < 0.01), as well as for LL2 (Estimate = 0.43, p < 0.001) and WL2 (Estimate = 0.08, p < 0.01). The population of Santa Lucía showed a significant negative effect on LLI (Estimate = -0.23, p < 0.05) and WLI (Estimate = -0.05, p < 0.05), indicating smaller length and width of the second femur in this population relative to Capital.

**Table 2:** Linear models for leg measurements of *T. infestans* urban and rural populations.

Predictors	LLI		WLI		LL2		WL2	
	Est.	S. E.	Est.	S. E.	Est.	S. E.	Est.	S. E.
(Intercept)	4.32 ^***^	0.06	0.83 ^***^	0.01	4.37 ^***^	0.06	0.82 ^***^	0.01
POP [CH]	-0.10	0.09	-0.03	0.02	-0.18	0.11	-0.02	0.02
POP [RI]	-0.00	0.08	0.01	0.02	-0.01	0.10	0.01	0.02
POP [RW]	-0.05	0.11	-0.03	0.02	-0.12	0.13	-0.01	0.03
POP [SL]	-0.23 ^*^	0.10	-0.05 ^*^	0.02	-0.16	0.10	-0.01	0.02
POP [VF]	0.37 ^***^	0.09	0.07 ^**^	0.02	0.43 ^***^	0.10	0.08 ^**^	0.02
SEX [M]	-0.06	0.08	-0.05 ^**^	0.02	-0.06	0.10	-0.02	0.02
POP [CH] × SEX [M]	-0.01	0.13	0.03	0.03	0.11	0.15	0.01	0.03
POP [RI] × SEX [M]	-0.18	0.12	0.01	0.03	-0.18	0.14	-0.03	0.03
POP [RW] × SEX [M]	0.02	0.14	0.04	0.03	0.09	0.17	-0.02	0.04
POP [SL] × SEX [M]	0.43 ^**^	0.16	0.07	0.04	0.37	0.18	0.02	0.04
POP [VF] × SEX [M]	-0.15	0.13	0.00	0.03	-0.19	0.15	-0.02	0.03
Observations	104		104		99		103	
R^2^/R^2^ adjusted	0.421/0.352		0.401/0.330		0.399/0.323		0.302/0.217	

Est., Estimates; S. E., standard error; POP, population; M, male; CA, Capital; CH, Chimbas; RI, Rivadavia; RW, Rawson; SL, Santa Lucía; VF, Valle Fértil; ^*^p<0.05. ^**^p<0.01. ^***^p<0.00.

Sex showed a significant negative effect only on WLI (Estimate = -0.05, p < 0.01), with males having narrower first femurs than females. Finally, limited significant interactions suggest that sex differences across populations are generally similar, with one exception, SL for LLI (Estimate = 0.43, p < 0.01). Thus, the difference in the length of the second femur in Santa Lucía between sexes is more pronounced compared to the other populations and with an opposite pattern than Capital (**Supplementary Material 5**).

### Colorimetric analyses

According to the majority rule, the best number of clusters to describe the wing color groups was two (7 indices out of 24). The same was true for connexivum color (12 out of 24). However, the clusters did not consistently correspond to any specific population group or sex. Individuals from all populations were included in both clusters for each analysis (results not shown).

## Discussion

We have found significant morphological differences between urban and rural *T. infestans* collected in the Province of San Juan. However, no chromatic differences were associated with urban or rural environments, nor with sex. Female forewings, pronota, heads and legs were smaller in highly urban environments compared to a rural population. A similar trend was observed for males, particularly for wing size. Sex differences in phenotypic plasticity are common, particularly in body size responses to environmental factors. In insects, females—often the larger sex—typically exhibit greater plasticity than males under conditions like food limitation, quality, and larval density, especially in species with pronounced sexual size dimorphism (54). However, sex differences in temperature-induced plastic responses do not consistently differ between sexes or correlate with sexual size dimorphism (55), pointing to a similar thermal response of development time in males and females (54). Urban heat islands (UHIs)—localized areas where temperatures are higher than surrounding rural regions due to human activities and built environments—can significantly impact animal populations in cities. For example, in our study areas, the maximum temperature in Gran San Juan ranges from 32°C in January to 17.1°C in July, while in Valle Fértil, the corresponding temperatures are 29.1°C in January and 15.1°C in July (37, 40). Elevated temperatures may alter species’ physiology, behavior, and life cycles, often favoring heat-tolerant or invasive species over native ones. For instance, thermoregulation demands can increase metabolic stress in some species, while others may benefit from longer active periods or altered reproduction timing (56, 57). A study on the bees *Halictus scabiosae* and *Osmia cornuta* and the wasp *Polistes dominula* along an urbanisation gradient in Milan, Italy, showed a reduction of the body size in hotter or low vegetation productivity sites (58). The authors propose that bees, as ectothermic insects, when exposed to elevated temperatures from the UHI effect may accelerate larval development, leading to smaller adults. Another possible explanation is that size reduction is an adaptation to urban heat, aligning with Bergmann’s rule, as smaller bodies with higher surface area-to-volume ratios lose heat more efficiently (59). These findings suggest that the smaller body sizes observed in *T. infestans* from urban environments may be influenced by the UHI effect, which likely accelerates development and favors phenotypes better adapted to elevated temperatures. This coincides with the trends reported for other ectothermic insects in urban settings, where thermal stress and altered developmental conditions drive morphological changes, potentially as a mechanism to enhance heat dissipation and improve survival in hotter, urbanized landscapes.

Schofield et al. (60) proposed that the characteristics that allow triatomine domiciliation are, among others, a simpler sensory system as habitat stability increases, a more relaxed bilateral symmetry, a general decrease in body size—mainly in females—leading to diminished sexual dimorphism, and a lower level of genetic variability. According to Flores-Ferrer et al. (61), only a small fraction of triatomine species is capable of establishing viable domestic populations, and this observation suggests that these particular species have traits enabling them to exploit domestic habitats. For example, Montes de Oca-Aguilar et al. (31) studied *T. dimidiata* in Yucatán State, Mexico and found a decrease in pronotum size and sensillum antennal density in urban individuals compared to their sylvatic and rural counterparts. The authors propose that this phenotypic simplification may indicate reduced flight or walking dispersal abilities in this species, likely linked to the predictable and limited host diversity in urban areas. Rural bugs exploit a broader host range, while urban bugs primarily feed on humans and domestic animals. *T. infestans*, a domestic species, is characterized by a high level of adaptation to human-modified habitats (62).

Our results of body size in San Juan domestic populations agree with the prediction of the simplification hypothesis of Schofield et al. (60), indicating that bugs collected in highly urbanized areas are smaller than those collected in rural zones of San Juan. Differently from what happens in *T. dimidiata* (31), this size reduction is generalized, affecting the heads, pronota, legs, and wings in the studied *T. infestans* populations. An exception is observed in individuals from the Department of Santa Lucía. Males collected there did not differ from rural individuals in wing size and showed a trend opposite to other populations in the length of the first leg, with males having larger femurs than females. The Department of Santa Lucía has recently become one of the most dynamic areas of the Gran San Juan, with the introduction of new and diverse functions in a traditionally agricultural zone (63). Until 1980, Santa Lucía exhibited linear urban growth along a west-east axis, guided by major communication routes. Urbanization also expanded northward but discontinuously, with isolated new neighborhoods reaching the border with the Department of Chimbas. The remaining departmental area was exclusively dedicated to agricultural activities. By 1990, the central area of the department became denser, and urbanization extended further east, a trend that has intensified over the past twenty years. Current urban expansion surpasses the central compact zone, with residential, industrial, commercial, and service developments emerging in the peripheral areas, replacing previously cultivated lands (63). The ongoing expansion of urbanized lots into previously rural areas may explain why the morphology of these insects more closely resembles that of rural *T. infestans* than their urban counterparts. However, due to the low sampling size available for Santa Lucía males, this pattern remains to be confirmed by higher samples.

We found not only size but also shape changes between urban and rural populations, particularly for wings and pronota. In the case of wings, the main directions of change according to the first two PCs were different from the axes of CVs (**Supplementary Materials 3** and **4**). Wing variation in PCA may reflect the lines of least resistance of morphology to evolution, showing the direction of the main variance within any population. In contrast, wing changes in CVA highlight more subtle shape changes by standardizing for within-group variance (64). Wing shape changes associated with urbanization have been observed in other insects. For instance, Multini et al. (65) in *Anopheles cruzii*, indicating that environmental changes driven by anthropization may significantly influence wing shape variation in this species. Another study in Brazilian populations of the native mosquito *Aedes scapularis* and the exotic species *Ae. albopictus* showed different levels of population structuring in wing shape for both species, suggesting different adaptive responses to urbanization (66). Regarding triatomines, a recent study of *T. garciabesi* and *T. guasayana* found wing shape changes associated with an anthropization gradient in the Argentine Dry Chaco in both species (67). On the contrary, Montes-de-Oca-Aguilar et al. (31) did not find any differences in wing shape among *T. dimidiata* individuals from urban, rural and silvatic landscapes from Mexico.

Wing shape differences, based on Mahalanobis distances between populations, were significant not only for urban-rural but also for several urban-urban comparisons. These results suggest that wing morphology may be influenced by complex and multifactorial processes. Developmental plasticity could play a role, as wing variation may respond to diverse environmental conditions beyond the rural-urban gradient. Additionally, flight demands might impose distinct selective pressures in urban environments, potentially driving changes in dispersal-related traits that are not fully captured by overall variation. Lastly, genetic drift could contribute to subtle differences in wing morphology among urban populations, reflecting random variation.

The pronotum, the dorsal plate of the prothorax (the first segment of the thorax in insects), often plays structural and protective roles. It serves as a robust platform for muscle attachment, which is crucial for movement and the interaction between the head, legs, and thorax. While the pronotum itself does not directly anchor the wings (which are connected to the mesothorax), it aligns the overall thoracic structure, maintaining balance and efficiency during flight.

The similarity between shape changes of the pronotum along the first two CVs and the first two PCs confirms that the largest axes of variation among specimens in the total sample are related to significant population differences in mean shape (**Supplementary Materials 3**, **4**). In both males and females, Valle Fértil pronota tended to be shorter along the anteroposterior axis and broader in the humeral area compared to urban populations, suggesting a possible adaptation or relaxation of selection in urban environments. Interestingly, Fiad (68) observed opposite morphological changes in the pronotum of *T. garciabesi* and *T. guasayana*. In sites with high human disturbance, males and females exhibited broader pronota in the humeral area. The author relates these changes to the development of flight muscles and dispersal abilities (69): a higher thorax could harbor more developed fly muscles, indicating higher dispersal in more urbanized areas. For *T. infestans* in San Juan, it might suggest the opposite pattern, less flight dispersal in urban areas. This hypothesis agrees with results from the urban population of the species from Arequipa, Peru, where genetic studies indicate that insect dispersal is limited by streets (70).

Head shape changes were less significant in rural-urban population comparisons than in wings and pronote showing some differences among urban females. Several studies have demonstrated that the triatomine head is a highly plastic structure, exhibiting differences under diverse environmental and feeding conditions. For example, Nattero et al. (71, 72) observed phenotypic plasticity in head size and shape in guinea pig-fed *T. infestans* under both natural and experimental conditions. Similarly, Lunardi et al. (73) reported head shape differences in *T. williami* when fed with mammalian versus avian blood. A study on *R. ecuatoriensis* populations identified elongated heads in northern, predominantly palm-dwelling populations, in contrast to the shorter, stouter heads of southern Andean populations (33). The authors hypothesized that these morphological differences might be associated with a transition from palm habitats to vertebrate nest microhabitats. Finally, a study on *T. brasiliensis* detected differences in head shape between sylvatic and domestic and peridomestic populations, possibly indicating adaptations to different habitats (74). As in the case of wings, our results may indicate that head shapes are affected by other sources of variation besides urban and rural environments, particularly in females.

Most insects display colors by absorbing or reflecting sunlight through pigments, cuticular surface structures, or a combination of both. This diversity of colors serves a range of functions, like body protection, signaling, mating, thermoregulation and UV resistance (75). Previous studies found chromatic variation in triatomines, both in sylvatic (76) as in domestic and peridomestic environments (51, 77, 78). However, our clustering analyses for wing and connexivum color did not align consistently with population groups or sex, suggesting that color variation is not strongly associated with these factors in San Juan populations.

Finally, we would like to point out some considerations regarding our study. First, the possibility that, in addition to urbanization, size differences between individuals from the urban populations and Valle Fértil may result from climatic differences. A previous study of 21 populations of *T. infestans* found an association between wing size and certain temperature and precipitation variables (30). This remains an aspect to explore in future research to determine whether these small-scale differences can influence morphology in the same way as at a broader geographical level. Second, there was a sampling imbalance, with 87 urban specimens from five populations compared to 18 rural specimens from a single population. We addressed this by conducting all analyses at the population level, where sampling was more balanced. Every pairwise comparison between the rural population and each urban population revealed significant morphometric differences. However, as only one rural site was included, broader conclusions regarding rural environments should be interpreted with caution.

Although some urban and rural *T. infestans* populations have been characterized in San Juan in the present work, the morphology of potential sylvatic foci remains entirely unknown due to their absence in local surveys. This represents a significant gap in our understanding of the species’ full phenotypic range and evolutionary trends in the region. We are actively pursuing collaborations to expand sampling efforts in sylvatic areas where *T. infestans* might persist. Adaptation of vector insects to urban environments, particularly in a world where urbanization is rapidly increasing alongside climate change, poses a significant challenge to vector control programs. As mentioned earlier, Chagas is a complex socio-environmental phenomenon. Vector control programs that must address urban problems face enormous challenges, linked to environmental, social, political and economic factors that further complicate control efforts. Social challenges include high population densities inhabiting residences in close proximity, which can be both horizontal and vertical building structures. From a political perspective, the absence of coordinated policies across governmental levels can hinder program effectiveness. Economically, limited financial resources can also strain control efforts. Addressing these interconnected challenges requires multisectoral, sustainable approaches that integrate epidemiological, environmental, social, economic, and political perspectives into vector control strategies in urban settings. For example, current Chagas control programs in Argentina heavily rely on protocols designed for rural environments and the insects that inhabit them. Recently, an inter-institutional consortium has been established, with the Argentine Ministry of Health, provincial health authorities, research groups and collaborators from Brazil and Uruguay, in order to assess the current state of urban Chagas and identify necessary changes to improve its control in cities (Mesa de Trabajo de Chagas Urbano 2021 (42)). Many questions arose, such as whether bugs have changed during the process of urbanization and how far they disperse within these new urban settings. Our findings indicate that urban bugs differ in size and shape from their rural counterparts, and these changes may be associated with adaptations to urban life and changing dispersal patterns. Additionally, some differentiation was observed between departments, suggesting varying epidemiological and/or microenvironmental characteristics among them.

Despite the brutal government cuts on funding of Argentine science and health institutions (79–81), we hope to continue with more studies of Chagas in urban areas, particularly those based on genetic markers. They will provide insights into the genetic diversity, population structure, and evolutionary pathways of urban Chagas vectors, shedding light on how these insects adapted to urban landscapes and providing valuable information to improve control strategies.

## Data Availability

The raw data supporting the conclusions of this article will be made available by the authors, without undue reservation.
