# Supplementary material for: Where you live shapes who you are: morphological changes in urban Triatoma infestans
Source: Front Insect Sci. 2025 Jun 2;5:1593921. doi: 10.3389/finsc.2025.1593921 (PMC12171112; doi:10.3389/finsc.2025.1593921)
Supplement: Supplementary file 1 [file Table1.docx]

| Department | Environment | N° ♀ | N° ♂ |
| --- | --- | --- | --- |
| Capital | urban | 13 | 12 |
| Chimbas | urban | 7 | 9 |
| Rawson | urban | 5 | 9 |
| Rivadavia | urban | 10 | 11 |
| Santa Lucía | urban | 8 | 3 |
| Valle Fértil | rural | 9 | 9 |
| Total |  | 52 | 53 |
|  |  |  |  |

**Supplementary Material 1.** Number of females and males of *T. infestans* collected in urban and rural populations of San Juan.
