# Supplementary figures and images for: Where you live shapes who you are: morphological changes in urban Triatoma infestans
Source: Front Insect Sci. 2025 Jun 2;5:1593921. doi: 10.3389/finsc.2025.1593921 (PMC12171112; doi:10.3389/finsc.2025.1593921)

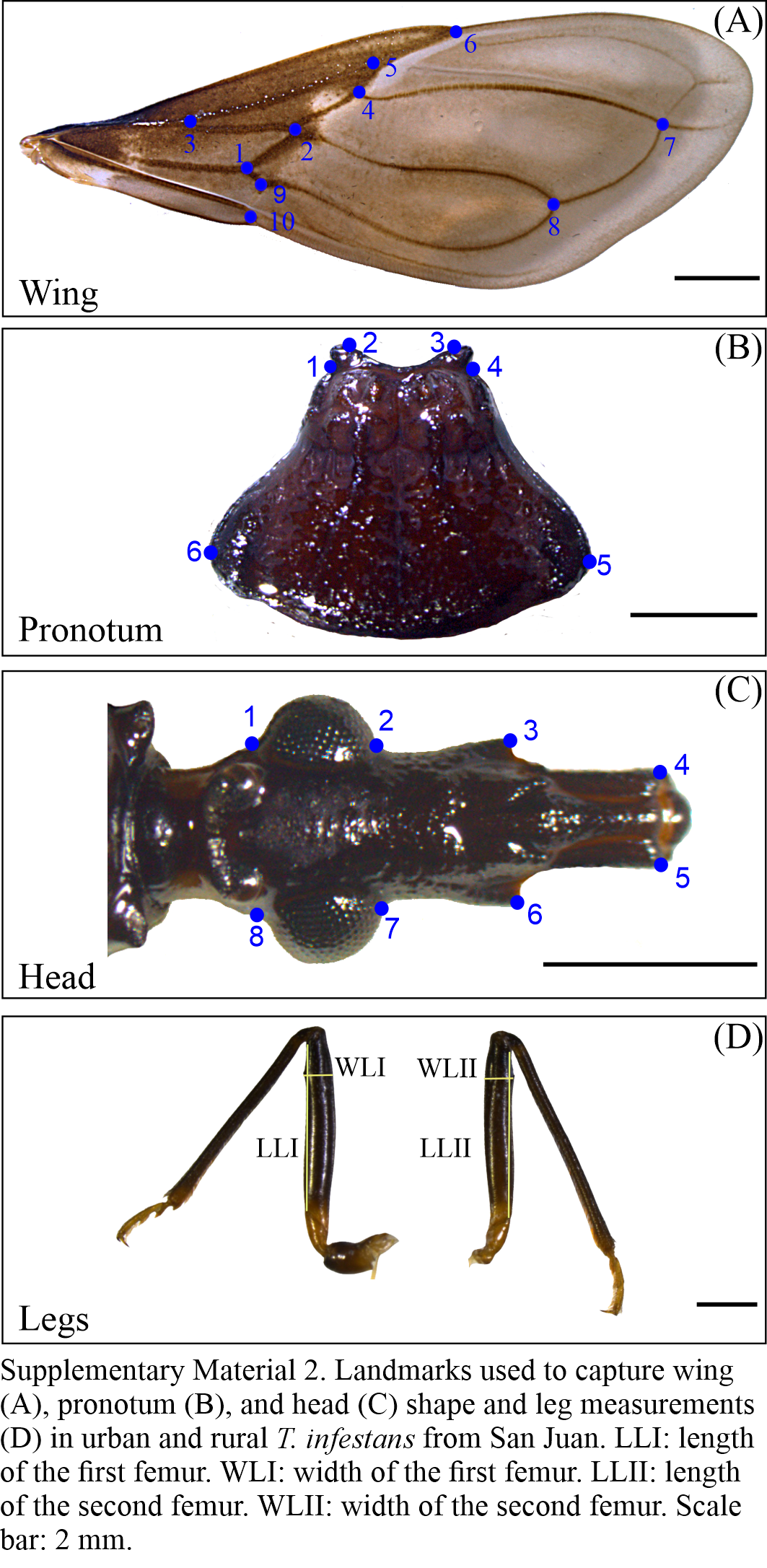

Supplement: Supplementary file 2 [file Image2.tif]

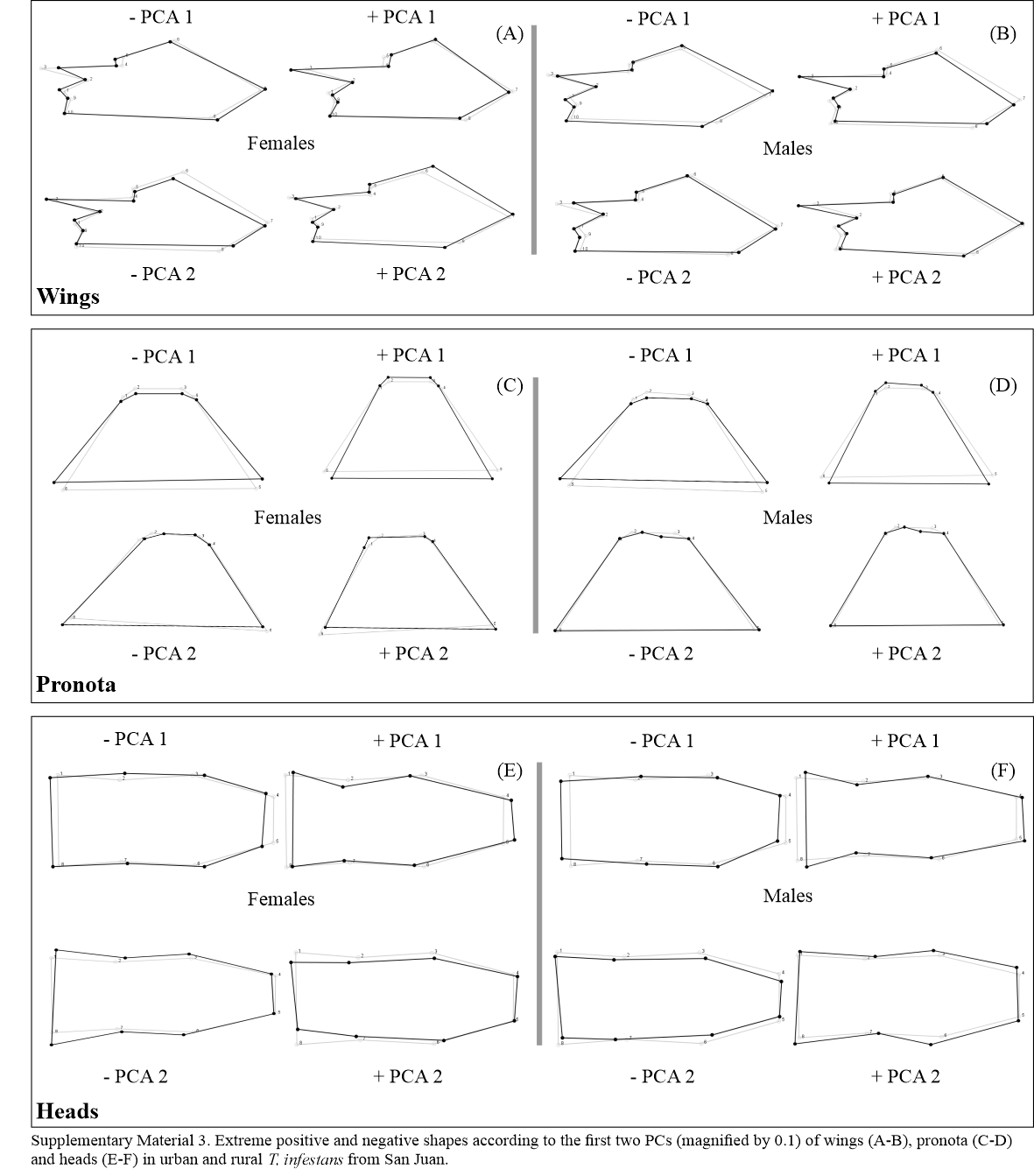

Supplement: Supplementary file 3 [file Image3.tif]

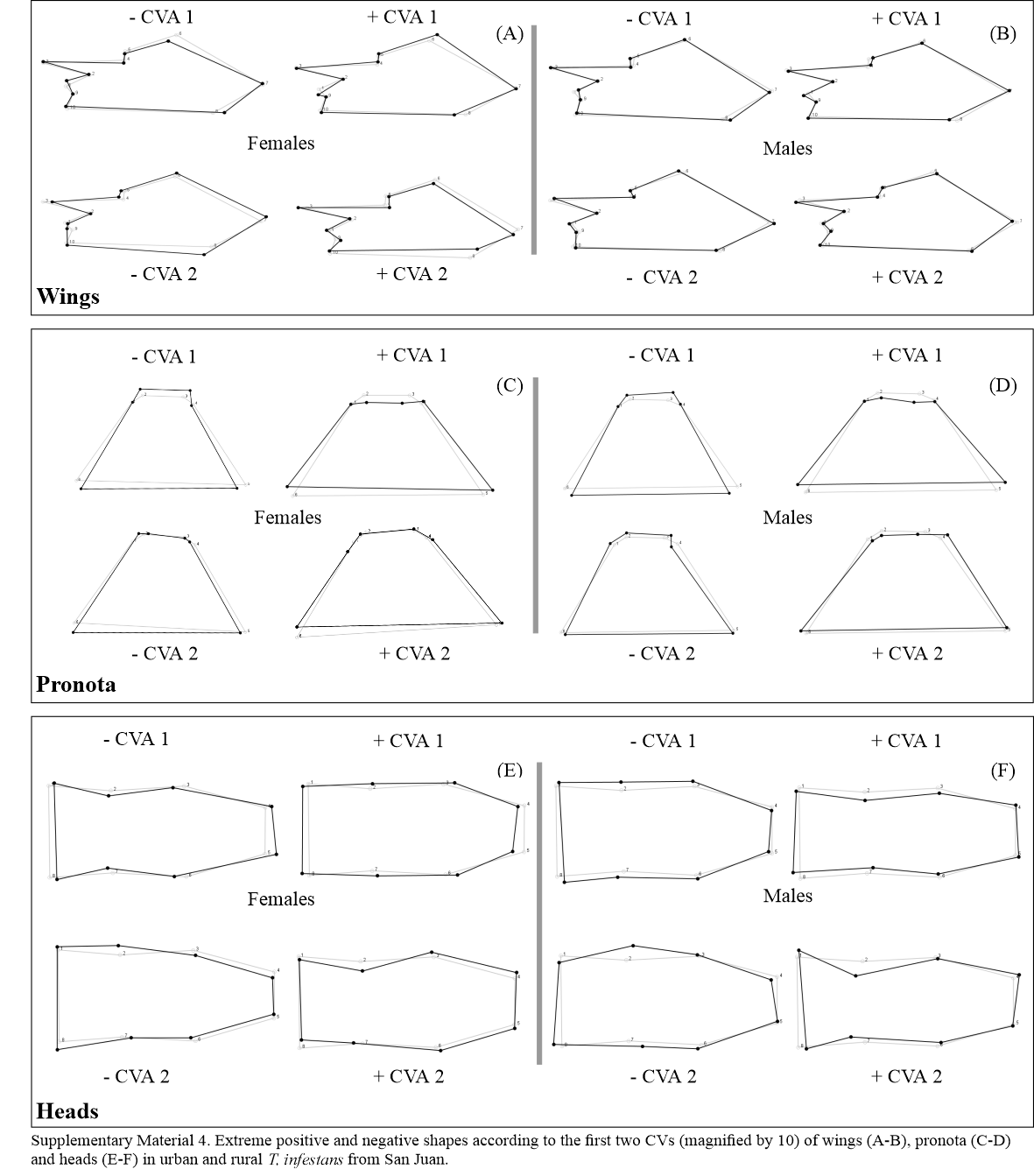

Supplement: Supplementary file 4 [file Image4.tif]

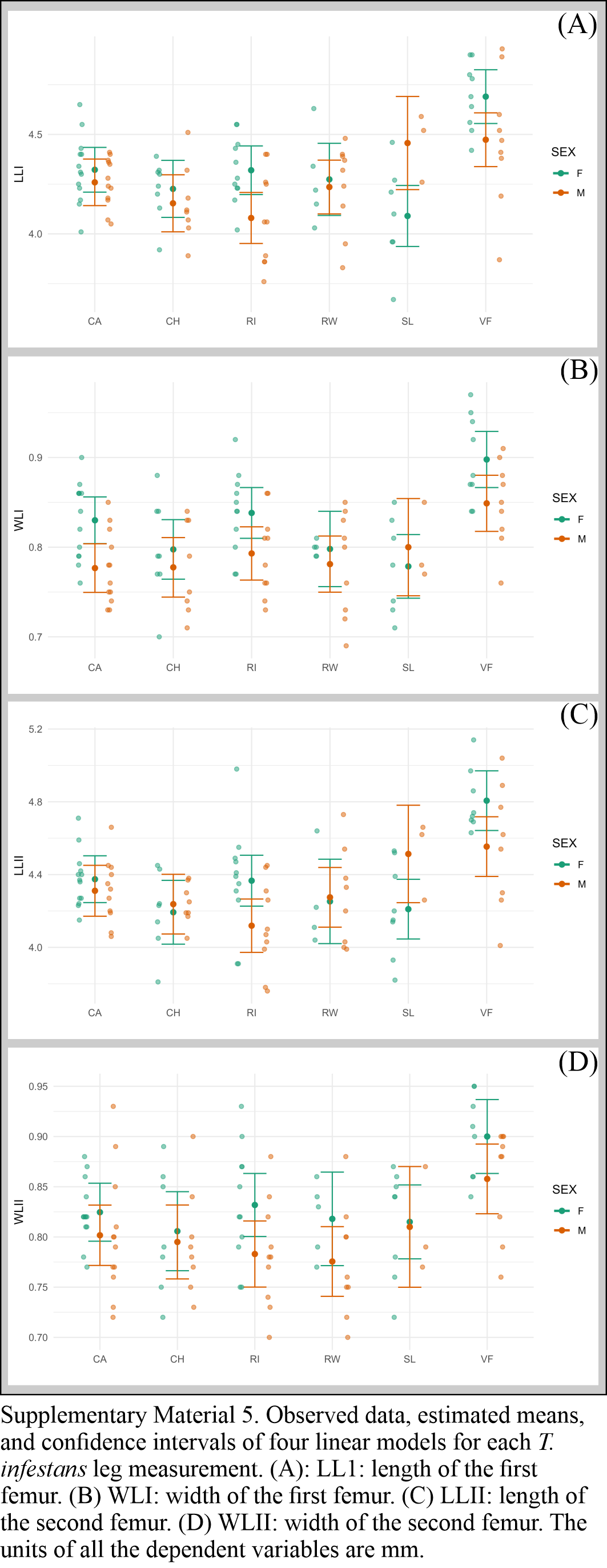

Supplement: Supplementary file 5 [file Image5.tif]
